# Circulating Clusterin and Osteopontin Levels in Asthma and Asthmatic Pregnancy

**DOI:** 10.1155/2017/1602039

**Published:** 2017-10-23

**Authors:** Brigitta Dombai, István Ivancsó, András Bikov, Dóra Oroszi, Anikó Bohács, Veronika Müller, János Rigó, Barna Vásárhelyi, György Losonczy, Lilla Tamási

**Affiliations:** ^1^Department of Pulmonology, Semmelweis University, Budapest, Hungary; ^2^Klinik für Anästhesie, Kantonsspital Aarau, Aarau, Switzerland; ^3^1st Department of Obstetrics and Gynecology, Semmelweis University, Budapest, Hungary; ^4^Department of Laboratory Medicine, Semmelweis University, Budapest, Hungary

## Abstract

Asthma in pregnancy poses a risk of adverse outcomes. Osteopontin and clusterin emerged as asthma biomarkers; however, their circulating levels during pregnancy are unknown yet. This cross-sectional study investigated peripheral osteopontin and clusterin levels and their relationship to disease control in 26 asthmatic pregnant (AP), 22 asthmatic nonpregnant (ANP), and 25 healthy pregnant (HP) women and 12 healthy controls (HNP). Osteopontin levels of ANP and HNP were similar (2.142 [1.483–2.701] versus 2.075 [1.680–2.331] ng/mL, *p* = 0.7331). Pregnancy caused a marked elevation in both healthy (HP: 3.037 [2.439–4.015] ng/ml, *p* = 0.003 versus HNP) and asthmatic (AP: 2.693 [1.581–3.620] ng/ml) patients; thus the pregnant groups did not differ (*p* = 0.3541). Circulating clusterin levels were comparable in ANP and HNP (109.2 [95.59–116.3] versus 108.8 [97.94–115.3] *µ*g/mL, *p* = 0.8730) and the level was lower in HP (98.80 [84.26–105.5] *µ*g/mL, *p* = 0.0344 versus HNP). In contrast, the level was higher in AP (111.7 [98.84–125.6] *µ*g/mL, *p* = 0.0091 versus HP). In ANP, a positive correlation of PEF (*r* = 0.3405; *p* = 0.0221) and a negative correlation of *R*_aw_ (*r* = −0.3723; *p* = 0.0128) to clusterin level were detected. Circulating osteopontin level increases in pregnancy regardless of concomitant well-controlled asthma, indicating its gestational role. Clusterin level decreases in healthy but not in asthmatic pregnancy and correlates directly with lung function.

## 1. Introduction

Asthma is one of the most common chronic diseases complicating pregnancy [1, 2], occurring in 8–12% of all gestations [3]. It represents a risk of potentially serious fetal and maternal morbidities, such as gestational hypertension, low birth weight, preeclampsia, preterm delivery, and increased neonatal mortality [4, 5]. On the other hand, pregnancy may also influence the control of asthma, leading to the deterioration of symptoms in one-third of pregnant women [6]. It is important to note that proper disease control decreases the risk of adverse pregnancy outcomes [7]; thus the maintenance of optimal asthma control and identification of pregnant women at risk of uncontrolled asthma are extremely important. However, treating asthma during pregnancy is a serious challenge for specialists due to the alterations in immune phenotype [8] and cytokine patterns [9] and the fact that pregnancy itself can influence spirometry results [10]. Unfortunately, the available techniques used to determine specific asthma phenotypes (e.g., induced sputum) are semi-invasive, and thus they should be avoided during pregnancy. Therefore, it would be crucial to have clinically usable biomarkers that can be linked to the loss of asthma control, which might help to detect the elevated risk of complications. However, easily obtainable peripheral blood biomarkers related to deteriorating disease control during asthmatic pregnancy are still not available.

Osteopontin is a multifunctional extracellular matrix protein and immune modulator originally discovered in bone but later identified in many cell types, such as bronchial epithelial cells and inflammatory cells around the airways, including T-cells and eosinophils [11–16]. It has a secreted soluble and an intracellular nonsecreted form. As a secreted acidic glycoprotein [16], it acts both as an extracellular matrix protein and as a cytokine [17], which plays a role in the migration of a number of inflammatory cells, including monocytes [18], lymphocytes [19], and neutrophils [20–22]. Although osteopontin has been recognized as a key cytokine involved in Th1 cell dominant immune responses, recent findings demonstrate that osteopontin levels are also elevated in Th2 cell related conditions, such as asthma and IgE-mediated allergic diseases. The exact role of osteopontin in allergy is not fully elucidated, but there are both human-model and animal-model studies that strongly support the hypothesis that osteopontin is an effector involved in critical steps of allergic inflammation [11]. Elevated osteopontin levels were demonstrated also in the sputum and bronchoalveolar lavage fluid (BALF) of asthmatic patients compared to the samples of healthy test subjects, and in these studies the BALF osteopontin levels showed correlation with the peripheral eosinophil count [22, 23].

During pregnancy, osteopontin has diverse biological functions in the uterus from the peri-implantation period till the end of gestation. It is thought to play a role in the uteroembryonal synchronization and is associated with the blastocysts' implantation competency [24, 25]. The rising level of osteopontin during pregnancy and its relation to the number of recruited decidual NK cells [26] suggest that it has an important role in the maintenance of a successful pregnancy. This idea is supported even further by some studies that detected lower osteopontin levels in pregnant women with preeclampsia and unexplained recurrent miscarriage [26, 27].

Clusterin is a multifunctional, stress-induced, ATP-independent molecular chaperone, expressed in most tissues and human fluids analyzed [28, 29]. It has two isoforms: a precursor nuclear form and a secreted extracellular form [29]; the two forms have been shown to have opposite functions. The nuclear form is thought to be associated with the promotion of cell death and apoptosis, while the secreted form is thought to be upregulated as a protective response during oxidative stress and endothelial injury [29]. The expression of clusterin is increased in several diseases, including diabetes, atherosclerosis, and Alzheimer's disease [30]. It takes part in the inhibition of complement-membrane attack complex, protection against inflammation, and cytoprotection at fluid tissue boundaries [29]. Secreted clusterin is involved in cancer progression, because, as a chaperon, it protects cells from varied therapeutic stressors that induce apoptosis, including radiation, cytotoxic chemotherapy, and biologic agents [28]. Extracellular clusterin exerts potent effects on the formation of amyloid in vitro, and it is suggested that in some circumstances increasing in vivo clusterin levels could be a therapeutic tool in extracellular protein deposition disorders [31]. As Carnevali et al. demonstrated, it may have a protective role against oxidative stress in the airways of smokers [32]. Circulating clusterin concentration was found to be elevated in patients with severe asthma, and it showed an inverse correlation with lung function; however, clusterin was considered an indicator of oxidative stress rather than a protective factor [30]. It also correlated with the age of the patients and showed a significant decrease in steroid-naïve patients with the initiation of ICS therapy [30]. Regarding pregnancy, it may be a part of pregnancy-related immune tolerance. Clusterin levels increase in preeclampsia, even prior to onset, so it may also serve as a predictive marker [33].

In the present study, we aimed to investigate osteopontin and clusterin levels in asthma and asthmatic pregnancy in comparison with healthy nonpregnant control subjects and healthy pregnant women. The possible relationships between osteopontin, clusterin, and asthma control determinants were also evaluated.

## 2. Materials and Methods

### 2.1. Ethics Statement

Written informed consent was obtained from all subjects, and our study was reviewed and approved by an independent ethical committee of the institution (Institutional and Regional Research Ethics Committee of Semmelweis Medical University). Laboratory studies and interpretations were performed on coded samples lacking personal and diagnostic identifiers. The study adhered to the tenets of the most recent revision of the Declaration of Helsinki.

### 2.2. Study Participants

In this cross-sectional study, 26 asthmatic pregnant (AP) and 22 asthmatic nonpregnant (ANP) women together with 25 healthy pregnant (HP) women and 12 healthy controls were enrolled. Asthmatic patients were recruited at their regular visit at the outpatient clinic of the Department of Pulmonology, Semmelweis University. They had persistent disease and they were diagnosed according to the current guidelines (GINA; [1]). Exclusion criteria included the existence of any other chronic disease (except for allergic rhinitis), multifetal gestation, maternal or fetal infections within six weeks of measurement, and current smoking (or more than 5-pack-year of smoking history). The patients were asked not to use their medications 12 hours before their visits. Healthy pregnant subjects were enlisted when attending their scheduled visit (mostly in the third trimester of pregnancy) at the 1st Department of Obstetrics and Gynecology, Semmelweis University. Healthy, nonpregnant controls were volunteer blood donors, with a negative history and negative status upon detailed physical and routine laboratory examination.

### 2.3. Laboratory Procedures

Plasma osteopontin and clusterin concentrations were measured with the Human Osteopontin (OPN) Quantikine ELISA Kit (RD-DOST00) and with the Human Clusterin Quantikine ELISA Kit (RD-DCLU00), respectively (R&D Systems, Inc., Minneapolis, USA), at the Central Laboratory of Department of Laboratory Medicine. Plasma was isolated from EDTA anticoagulated blood samples between 3 and 8°C within 30 minutes after sampling and stored at −80°C until measurement as recommended by the manufacturer.

### 2.4. Lung Function Measurement and Asthma Control Evaluation

Lung function tests were performed by means of electronic spirometer (PDD-301/s, Piston, Budapest, Hungary), according to the American Thoracic Society (ATS) guidelines [34]. After three technically acceptable manoeuvres, we were using the one with the best results. Forced expiratory volume in one second (FEV_1_), peak expiratory flow (PEF), and airway resistance (*R*_aw_) were measured. Asthma control was assessed using the asthma control test (ACT), suggested by the current (GINA) guidelines [1].

### 2.5. Statistics

Data distribution was analyzed by D'Agostino-Pearson normality test. Comparisons between the study groups were made with Kruskal-Wallis and Dunn's post hoc multiple comparison tests. Correlation analyses were performed using Spearman's test due to nonnormal distribution of data. Area under the curve (AUC) values of receiver operating characteristics (ROC) curves were calculated using standard methods and data are presented as AUC-ROC (95% CI). *p* values < 0.05 were considered significant. Statistics were calculated using the GraphPad Prism software 6 (GraphPad Software, La Jolla, CA, USA). Data are expressed as median [interquartile range]. Sample size was estimated to find relevant differences (effect size of 0.40 and power of 0.80) among the four groups in case of the two examined mediators (osteopontin and clusterin) with respect to the asymptotic relative efficiency of nonparametric tests.

## 3. Results

### 3.1. Clinical Characteristics

All of the gathered clinical and demographical data and the measured inflammatory parameters of the subjects are summarized in Table 1. Ages of participants were comparable among the four groups. Gestational age at blood sampling was lower in the AP than in the HP group (24.5 [19–34.25] versus 35 [32.5–37] weeks, *p* = 0.0002). Gestational age at delivery and fetal birth weight were comparable in the two pregnant groups.

No difference was detected either in the severity or control of asthma or the daily dose of inhaled corticosteroids (ICS) between the ANP and AP groups (Table 1). 11 asthmatic nonpregnant and 17 asthmatic pregnant patients received ICS treatment, while 11 patients in the ANP group and 9 patients in the AP group were steroid-naïve. Steroid-naïve and steroid-treated patients did not differ in any of the investigated parameters (such as spirometry results, ACT total scores, inflammatory parameters, and peripheral eosinophil cell counts). ACT total scores (24 [20.5–25]^*n*=21^ in the ANP and 22 [19–25]^*n*=24^ in the AP group) showed acceptable and similar levels of disease control of the patients in both asthmatic groups. 2 ANP and 3 AP patients were nonallergic asthmatics; all the other asthmatic patients suffered from allergic/atopic disease.

### 3.2. Comparison of Circulating Marker Levels among the Four Groups

**Figure 1:**
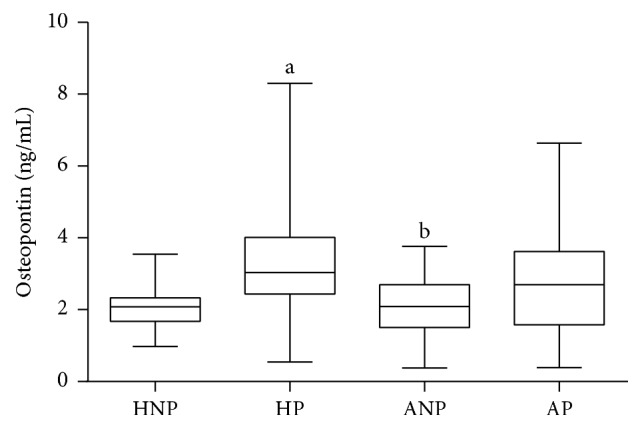
*Circulating osteopontin levels in healthy nonpregnant and pregnant and in asthmatic nonpregnant and pregnant subjects* (data are expressed as median [interquartile range]). HNP: healthy nonpregnant; HP: healthy pregnant; ANP: asthmatic nonpregnant; AP: asthmatic pregnant. ^a^*p* < 0.05 versus HNP; ^b^*p* < 0.05 versus HP.

Pregnancy per se caused elevation in circulating osteopontin level, as it was significantly higher in the HP than in the HNP group (3.037 [2.439–4.015] versus 2.075 [1.680–2.331] ng/mL, *p* = 0.0036). In asthmatic subjects, this difference only reached the level of a trend (2.693 [1.581–3.620] in AP versus 2.086 [1.508–2.697] ng/ml in ANP, *p* = 0.0850). Osteopontin levels of the two pregnant groups did not differ (3.037 [2.439–4.015] ng/mL in HP versus 2.693 [1.581–3.620] ng/mL in AP, *p* = 0.3541), and the data of ANP and HNP subjects were similar as well (2.142 [1.483–2.701] versus 2.075 [1.680–2.331] ng/mL, *p* = 0.7331, Figure 1).

**Figure 2:**
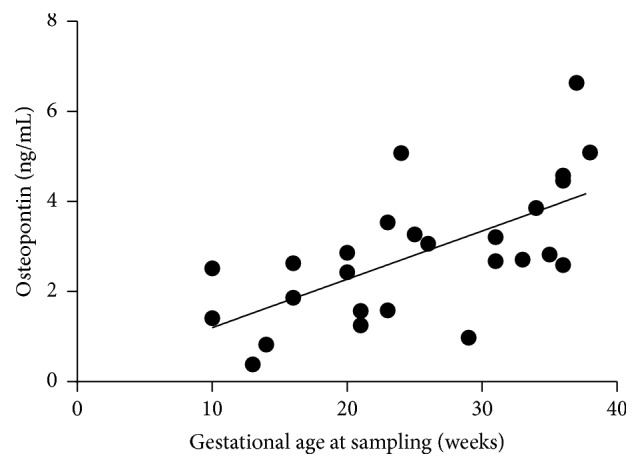
*Positive correlation of gestational weeks at sampling to circulating osteopontin in asthmatic pregnant patients. p* < 0.0001; *r* = 0.6924.

Circulating osteopontin correlated positively with the gestational age at sampling in the AP group (*r* = 0.6921; *p* < 0.0001; Figure 2).

**Figure 3:**
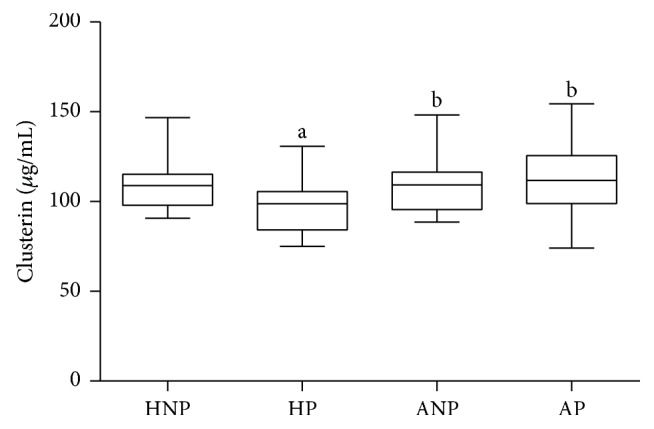
*Circulating clusterin levels in healthy nonpregnant and pregnant and in asthmatic nonpregnant and pregnant subjects (data are expressed as median [interquartile range]).* HNP: healthy nonpregnant; HP: healthy pregnant; ANP: asthmatic nonpregnant; AP: asthmatic pregnant. ^a^*p* < 0.05 versus HNP; ^b^*p* < 0.05 versus HP.

Asthma itself also did not influence circulating clusterin level, as ANP and HNP groups had similar results (109.2 [95.59–116.3] versus 108.8 [97.94–115.3] *µ*g/mL, *p* = 0.8730). On the other hand, circulating clusterin levels were significantly lower in the HP than in the HNP group (98.80 [84.26–105.5] versus 108.8 [97.94–115.3] *µ*g/mL, *p* = 0.0344). Since this pregnancy-specific decrease was absent in AP group (AP 111.7 [98.84–125.6] versus ANP 109.2 [95.59–116.3] *µ*g/mL), we could demonstrate a significant difference between the clusterin levels of the two pregnant groups (111.7 [98.84–125.6] *µ*g/mL in AP versus 98.80 [84.26–105.5] *µ*g/mL in HP, *p* = 0.0091; Figure 3 and Table 1).

**Figure 4:**
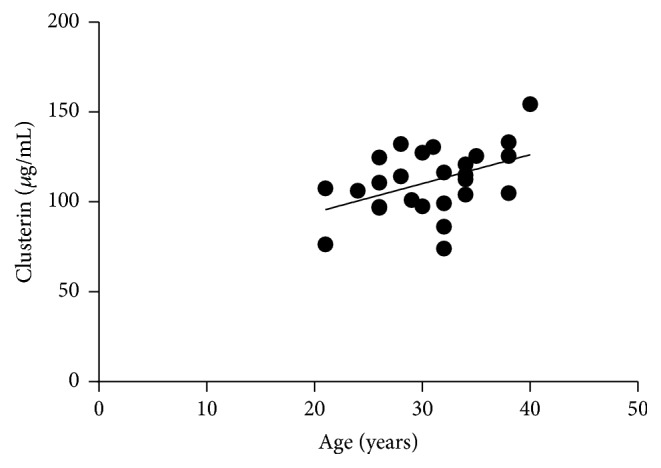
*Positive correlation of subjects' age to circulating clusterin levels in asthmatic pregnant patients. p* = 0.0442; *r* = 0.3978.

Circulating clusterin level correlated positively with the age of the patients in the AP group (*p* = 0.0442; *r* = 0.3978; Figure 4).

### 3.3. Relationship of Inflammatory Markers to Asthma Control Determinants and Obstetrical Data

**Figure 5:**
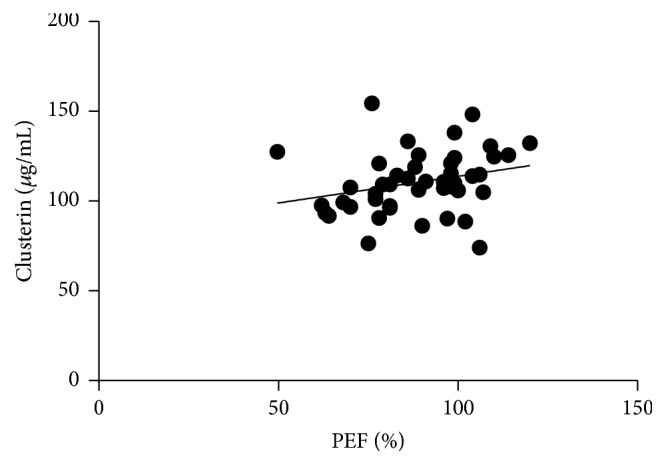
*Positive correlation of PEF and circulating clusterin levels in asthmatic patients (pregnant and nonpregnant). p* = 0.0221; *r* = 0.3405.

**Figure 6:**
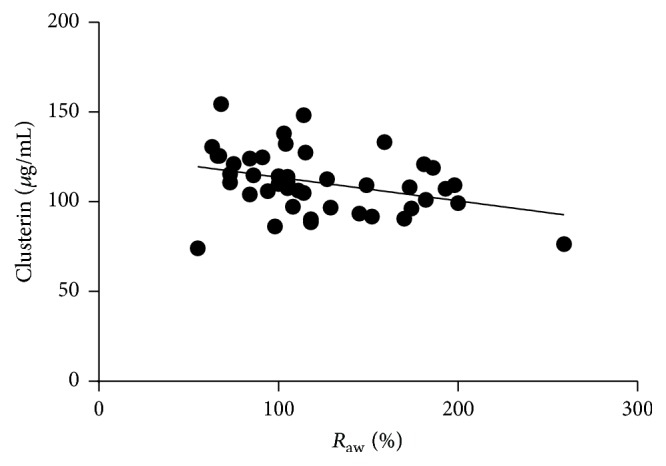
*Negative correlation of R*_aw_ *and circulating clusterin levels in asthmatic patients (pregnant and nonpregnant). p* = 0.0128; *r* = −0.3723.

For spirometry results, we observed a positive correlation between PEF and circulating clusterin levels (*r* = 0.4487; *p* = 0.0413; Figure 5) and a negative correlation in case of *R*_aw_ and circulating clusterin (*r* = −0.4764; *p* = 0.0290; Figure 6) levels in the ANP group. This association remained significant even when we merged the AP and ANP groups and investigated it in the whole asthmatic cohort (PEF: *r* = 0.3405 and *p* = 0.0221; *R*_aw_: *r* = −0.3723 and *p* = 0.0128). There were no correlations observed between any of the investigated inflammatory markers and the obstetrical parameters in the pregnant groups. Besides that, circulating osteopontin and clusterin levels were not influenced by the daily inhaled corticosteroid dose of the patients.

As current guideline [1] defines uncontrolled asthma as a disease with PEF below 80% predicted or ACT total score below 20, ROC analyses of asthmatic patients' data were performed in case of both mediators in subgroups of AP and ANP with PEF above and below 80% and ACT total score above and below 20. However, these analyses did not yield any statistically significant results.

## 4. Discussion

The aim of this study was to assess plasma osteopontin and clusterin levels in asthmatic pregnancy together with healthy and asthmatic nonpregnant and healthy pregnant subjects. Furthermore, we examined any possible relationships between these markers and asthma control determinants. We demonstrated an increase of osteopontin level in both pregnant groups compared with the nonpregnant respective ones; however, in asthmatic pregnancy, no further elevation was detected compared to healthy pregnancy. Thence, concomitant mostly well-controlled asthma did not influence circulating osteopontin level during pregnancy. Regarding circulating clusterin level, we observed a decrease in healthy pregnancy; however, this pregnancy-specific decrease was absent in asthmatic pregnant women. Furthermore, a positive correlation was detected between PEF and clusterin, and a negative correlation was detected between *R*_aw_ and clusterin levels in asthmatic nonpregnant patients.

The most important limitation of our study was the small sample size. Furthermore, our data may be confounded by the well-controlled state of nearly all enrolled asthmatic patients and thus may not represent a general asthmatic population. The reason for this is our clinical goal to achieve the best control of the disease (especially in pregnant patients). Nevertheless, our data must be considered with caution due to these limitations.

Osteopontin emerged as a promising inflammatory biomarker because of its multifunctional role in a wide range of inflammatory processes. It functions as an extracellular matrix protein and immune modulator, expressed by many cell types, such as bronchial epithelial cells, T-cells, dendritic cells, and eosinophils [11–16]. A number of studies demonstrated the importance of osteopontin in the development of several inflammatory diseases, particularly in connection with CD4+ T-helper-associated immune responses [11]. Firstly, it was identified as a key element of Th1 responses, inducing IL-12 production in macrophages and dendritic cells in vitro. The dendritic cells activated by osteopontin were shown to stimulate naïve T-cells to produce Th1-polarized cytokines. Osteopontin is involved in the pathogenesis of several diseases associated with Th1 responses, including rheumatoid arthritis, sarcoidosis, tuberculosis, Crohn's disease, and multiple sclerosis [11]. Recently, some studies investigated its role in bronchial asthma and allergic diseases. Compared to healthy control subjects, higher levels of osteopontin were measured in serum, sputum, and BALF of asthmatic patients [13, 14], and the differences were shown to be even more explicit in case of smoking asthmatics [23]. According to Hillas et al. [35], smoking habit significantly affects sputum osteopontin levels in asthma. Increased osteopontin levels were also observed in allergic conjunctivitis [36] and allergic rhinitis [37]. However, in accordance with our results, the increase does not seem to correlate with the severity or control of asthma [22]. Studies of endobronchial biopsies of asthmatic patients found increased osteopontin levels and positive correlation between the tissue remodelling and the bronchial expression of osteopontin [17, 22]. There are controversial data about the relationship between osteopontin levels and eosinophilia; while Liu et al. reported elevated serum osteopontin levels in asthmatic patients [38], a recent review suggests that osteopontin rather appears to be related to a neutrophil asthma phenotype and indicates disease severity [39]. Although there are studies that showed elevated osteopontin levels in asthmatic patients, in our study, this increase was absent. This apparent contradiction between our data and some of the previous results may be based on the different levels of the patient's asthma control, as our patients were mostly well controlled, with ACT scores of 22 or higher, and due to the fact that they mostly have eosinophil/atopic asthma, which, based on the recent findings [39], shows less connection with the peripheral osteopontin levels. However, it must be stressed that one of the major limitations of our study is the lack of uncontrolled, symptomatic asthmatics. It also should be noted that most of the earlier studies measured osteopontin levels in sputum or bronchoalveolar lavage fluid of asthmatic patients, and less studies measured the serum levels.

On the other hand, osteopontin also has a wide variety of functions in pregnancy, including a role as a component involved in adhesion and signal transduction at the uterine-placental interface, regulating immune cell behavior and cytokine production and expression in the uterine stroma [40, 41]. It has an essential role in the maintenance of a successful pregnancy from the implantation of the blastocyst [24, 25] until the end of gestation [26, 27].

Our study was the first to evaluate osteopontin levels in asthmatic pregnancy. In line with earlier findings [26], we were able to demonstrate an elevation of osteopontin levels in healthy pregnant women, which correlated positively with gestational age. There was a detectable but not significant elevation in asthmatic pregnant women too, compared to asthmatic nonpregnant patients. The absent of statistical significance in the latter case is probably in connection with one of the limitations of this study, more precisely the fact that the gestational age at sampling in the AP group was lower than that in the HP group. However, the connection between osteopontin level and pregnancy suggests that osteopontin as an asthma biomarker must be handled with caution during pregnancy.

Clusterin is a sensitive cellular biosensor of oxidative stress [30] which is an important factor in the pathophysiology of asthma and also maternal asthma during pregnancy [42, 43]. The function of clusterin in oxidative stress has not been determined yet, but it is thought to protect cells from oxidative injury [30, 32]. Clusterin levels are elevated in many different pathological conditions, like apoptosis, hypoxia, or neurodegenerative conditions, and in several diseases, such as diabetes and atherosclerosis, and treatment recurrent cancers, including castrate resistant prostate cancer [28, 30, 44]. In line with earlier findings [30], we found a correlation between plasma clusterin levels and age in the asthmatic pregnant group. However, we could not confirm all the previous data [30], as we did not detect higher plasma clusterin levels in asthmatic patients. The reason of this difference could be the fact that, based on the almost physiological lung function results and CRP levels, together with ACT scores indicating nearly no symptoms, our asthmatic patients were mostly well controlled, while increased clusterin levels were mostly observed in patients with severe uncontrolled disease [30]. Furthermore, contrary to some previous findings, in which circulating clusterin levels showed inverse correlation with the pulmonary function [30], we observed a significant positive correlation of PEF and a negative correlation of *R*_aw_ to circulating clusterin levels in asthmatic, nonpregnant patients. This surprising result may also be the consequence of one of the major limitations of our study, which is the mostly well-controlled state of our patients, while in previous studies, patients were uncontrolled. In controlled patients, the dominant function of clusterin may be the anti-inflammatory, protective function [32]; however, data about clusterin are still scarce and its functions are not fully elucidated yet.

Furthermore, many studies have shown elevated serum and placenta clusterin levels in preeclamptic women [29, 33, 42, 43], elevation of which could be in connection with endothelial cell injury or oxidative stress [43]; however, serum clusterin level was never examined in asthmatic pregnancy before. In a recent study, the elevation of clusterin levels was observed prior to onset of preeclampsia, suggesting that it may also serve as a predictive marker of adverse pregnancy outcomes [33]. In our study, we found decreased plasma clusterin in healthy pregnant women, but this decrease was absent in the asthmatic pregnant group. This relative increase in asthmatic versus healthy pregnancy may be the consequence of enhanced oxidative stress, which characterizes asthma during pregnancy [42].

In conclusion, increase of osteopontin level in pregnancy independently of concomitant asthma suggests that it has an important role during gestation and indicates caution if used as a biomarker in pregnant state. Clusterin level decreases in healthy pregnant women; however, this decrease is absent in asthmatic pregnancy, which may be related to asthmatic inflammation. On the other hand, positive correlation of PEF and negative correlation of *R*_aw_ to circulating clusterin level are probably the result of its anti-inflammatory effect in well-controlled asthma; however, further research on circulating clusterin level as an asthma biomarker in controlled versus uncontrolled disease is warranted.

## Figures and Tables

**Table 1:** Clinical data and inflammatory parameters of the four study groups (median [interquartile range]).

	HNP (*n* = 12)	HP (*n* = 25)	ANP (*n* = 22)	AP (*n* = 26)
Age (years)	29.5 [26.25–37]	34 [29.5–35.5]	35 [28.5–38.25]	31.5 [26–34]

Gestational age at sampling (weeks)	NA	35 [32.5–37]	NA	24.5 [19–34.25]

Gestational age at delivery (weeks)	NA	39 [38–40]	NA	38 [37–39]^*n*=21^

Fetal birth weight (grams)	NA	3240 [2970–3730]	NA	3250 [2995–3745]^*n*=21^

FEV_1_ (% of predicted)	NA	NA	97 [92–108]^*n*=21^	94 [84.75–104.3]

PEF (% of predicted)	NA	NA	98 [81–99.5]^*n*=21^	84.50 [75.25–103.5]^*n*=24^

*R*_aw_ (% of predicted)	NA	NA	118 [97–171.5]^*n*=21^	105 [73–129]^*n*=23^

FEF 25–75% (% of predicted)	NA	NA	77 [63.5–93]^*n*=21^	83 [64–104]^*n*=23^

ACT total score	NA	NA	24 [20.5–25]^*n*=21^	22 [19–25]^*n*=24^

Daily dose of ICS (beclomethasone equivalent, *µ*g)	NA	NA	200 [0–1000]	650 [0–1000]

Eosinophil cells (%)	1.595 [1.260–2.478]	NA	2.420 [1.273–6.553]^*n*=14^	2 [1.218–3.060]^*n*=20^

IL-6 (pg/mL)	NA	NA	1.795 [1.500–2.278]^*n*=20^	1.935 [1.5–3.433]^*n*=22^

CRP (mg/mL)	3.0 [1.3–3.0]^*n*=11^	NA	3 [3–5]^*n*=21^	4.845 [3–8.750]^*n*=24^

Osteopontin (ng/mL)	2.075 [1.680–2.331]	3.037 [2.439–4.015]^a^	2.086 [1.508–2.679]^b^	2.693 [1.581–3.620]

Clusterin (*µ*g/mL)	108.8 [97.94–115.3]	98.80 [84.26–105.5]^a^	109.2 [95.59–116.3]^b^	111.7 [98.84–125.6]^b^

HNP: healthy nonpregnant; HP: healthy pregnant; ANP: asthmatic nonpregnant; AP: asthmatic pregnant; FEV_1_: forced expiratory volume in 1 second; PEF: peak expiratory flow rate; *R*_aw_: airway resistance; FEF_25–75%_: forced expiratory flow at 25–75% of forced vital capacity; ACT: asthma control test; ICS: inhaled corticosteroid; CRP: C-reactive protein; IL-6: interleukin-6; NA: not applicable. ^a^*p* < 0.05 versus HNP; ^b^*p* < 0.05 versus HP.

## References

[1] Global Initiative for Asthma, http://www.ginasthma.org, 2017

[2] Kwon H. L., Belanger K., Bracken M. B. (2003). Asthma prevalence among pregnant and childbearing-aged women in the United States: estimates from national health surveys. *Annals of Epidemiology*.

[3] Charlton R. A., Hutchison A., Davis K. J., de Vries C. S. (2013). Asthma Management in Pregnancy. *PLoS ONE*.

[4] Demissie K., Breckenridge M. B., Rhoads G. G. (1998). Infant and maternal outcomes in the pregnancies of asthmatic women. *American Journal of Respiratory and Critical Care Medicine*.

[5] Breton M.-C., Beauchesne M.-F., Lemière C., Rey É., Forget A., Blais L. (2009). Risk of perinatal mortality associated with asthma during pregnancy. *Thorax*.

[6] Murphy V. E., Clifton V. L., Gibson P. G. (2006). Asthma exacerbations during pregnancy: incidence and association with adverse pregnancy outcomes. *Thorax*.

[7] Tamási L., Horváth I., Bohács A., Müller V., Losonczy G., Schatz M. (2011). Asthma in pregnancy—immunological changes and clinical management. *Respiratory Medicine*.

[8] Toldi G., Molvarec A., Stenczer B. (2011). Peripheral T(h)1/T(h)2/T(h)17/regulatory T-cell balance in asthmatic pregnancy. *International Immunology*.

[9] Tamási L., Bohács A., Tamási V. (2010). Increased circulating heat shock protein 70 levels in pregnant asthmatics. *Cell Stress and Chaperones*.

[10] Grindheim G., Toska K., Estensen M.-E., Rosseland L. A. (2012). Changes in pulmonary function during pregnancy: A longitudinal cohort study. *BJOG: An International Journal of Obstetrics & Gynaecology*.

[11] Konno S., Kurokawa M., Uede T., Nishimura M., Huang S. (2011). Role of osteopontin, a multifunctional protein, in allergy and asthma. *Clinical & Experimental Allergy*.

[12] Nagasaka A., Matsue H., Matsushima H. (2008). Osteopontin is produced by mast cells and affects IgE-mediated degranulation and migration of mast cells. *European Journal of Immunology*.

[13] Takahashi A., Kurokawa M., Konno S. (2009). Osteopontin is involved in migration of eosinophils in asthma. *Clinical & Experimental Allergy*.

[14] Puxeddu I., Berkman N., Ribatti D. (2010). Osteopontin is expressed and functional in human eosinophils. *Allergy*.

[15] Kohan M., Bader R., Puxeddu I., Levi-Schaffer F., Breuer R., Berkman N. (2007). Enhanced osteopontin expression in a murine model of allergen-induced airway remodelling. *Clinical & Experimental Allergy*.

[16] Kurokawa M., Konno S., Matsukura S. (2009). Effects of corticosteroids on osteopontin expression in a murine model of allergic asthma. *International Archives of Allergy and Immunology*.

[17] Kohan M., Breuer R., Berkman N. (2009). Osteopontin induces airway remodeling and lung fibroblast activation in a murine model of asthma. *American Journal of Respiratory Cell and Molecular Biology*.

[18] Kon S., Yokosaki Y., Maeda M. (2002). Mapping of functional epitopes of osteopontin by monoclonal antibodies raised against defined internal sequences. *Journal of Cellular Biochemistry*.

[19] O'Regan A. W., Chupp G. L., Lowry J. A., Goetschkes M., Mulligan N., Berman J. S. (1999). Osteopontin is associated with T cells in sarcoid granulomas and has T cell adhesive and cytokine-like properties in vitro. *The Journal of Immunology*.

[20] Koh A., Da Silva A. P. B., Bansal A. K. (2007). Role of osteopontin in neutrophil function. *The Journal of Immunology*.

[21] Senger D. R., Perruzzi C. A., Papadopoulos-Sergiou A., Van De Water L. (1994). Adhesive properties of osteopontin: Regulation by a naturally occurring thrombin-cleavage in close proximity to the GRGDS cell-binding domain. *Molecular Biology of the Cell (MBoC)*.

[22] Samitas K., Zervas E., Vittorakis S. (2011). Osteopontin expression and relation to disease severity in human asthma. *European Respiratory Journal*.

[23] Samitas K., Zervas E., Xanthou G., Panoutsakopoulou V., Gaga M. (2013). Osteopontin is increased in the bronchoalveolar lavage fluid and bronchial tissue of smoking asthmatics. *Cytokine*.

[24] Xie Q., Qi Q., Chen Y., Xu W., Liu Q., Yang J. (2013). Uterine Micro-Environment and Estrogen-Dependent Regulation of Osteopontin Expression in Mouse Blastocyst. *International Journal of Molecular Sciences*.

[25] Chaen T., Konno T., Egashira M. (2012). Estrogen-Dependent Uterine Secretion of Osteopontin Activates Blastocyst Adhesion Competence. *PLoS ONE*.

[26] Qu X., Yang M., Zhang W., Liang L., Yang Y., Zhang Y. (2008). Osteopontin expression in human decidua is associated with decidual natural killer cells recruitment and regulated by progesterone. *In Vivo*.

[27] Xia J., Qiao F., Su F., Liu H. (2009). Implication of expression of osteopontin and its receptor integrin *ανβ*3 in the placenta in the development of preeclampsia. *Journal of Huazhong University of Science and Technology (Medical Sciences)*.

[28] Zoubeidi A., Chi K., Gleave M. (2010). Targeting the Cytoprotective Chaperone, Clusterin, for Treatment of Advanced Cancer. *Clinical Cancer Research*.

[29] Oztas E., Ozler S., Ersoy A. O. (2016). Increased levels of serum clusterin is associated with intrauterine growth restriction and adverse pregnancy outcomes in preeclampsia. *Journal of Perinatal Medicine*.

[30] Kwon H.-S., Kim T.-B., Lee Y. S. (2014). Clusterin expression level correlates with increased oxidative stress in asthmatics. *Annals of Allergy, Asthma & Immunology*.

[31] Yerbury J. J., Poon S., Meehan S. (2007). The extracellular chaperone clusterin influences amyloid formation and toxicity by interacting with prefibrillar structures. *The FASEB Journal*.

[32] Carnevali S., Luppi F., D'Arca D. (2006). Clusterin decreases oxidative stress in lung fibroblasts exposed to cigarette smoke. *American Journal of Respiratory and Critical Care Medicine*.

[33] Kolla V., Jenö P., Moes S., Lapaire O., Hoesli I., Hahn S. (2012). Quantitative proteomic (iTRAQ) analysis of 1st trimester maternal plasma samples in pregnancies at risk for preeclampsia. *Journal of Biomedicine and Biotechnology*.

[34] Miller M. R., Hankinson J., Brusasco V., Burgos F., Casaburi R. (2005). Standardisation of spirometry. *European Respiratory Journal*.

[35] Hillas G., Loukides S., Kostikas K. (2013). Increased levels of osteopontin in sputum supernatant of smoking asthmatics. *Cytokine*.

[36] Uchio E., Matsuura N., Kadonosono K., Ohno S., Uede T. (2002). Tear osteopontin levels in patients with allergic conjunctival diseases. *Graefe's Archive for Clinical and Experimental Ophthalmology*.

[37] Liu Y., Lu X., Yu H.-J. (2010). The expression of osteopontin and its association with Clara cell 10 kDa protein in allergic rhinitis. *Clinical & Experimental Allergy*.

[38] Liu W., Xia W., Fan Y. (2012). Elevated serum osteopontin level is associated with blood eosinophilia and asthma comorbidity in patients with allergic rhinitis. *The Journal of Allergy and Clinical Immunology*.

[39] Zissler U. M., Esser-von Bieren J., Jakwerth C. A., Chaker A. M., Schmidt-Weber C. B. (2016). Current and future biomarkers in allergic asthma. *Allergy*.

[40] Johnson G. A., Burghardt R. C., Bazer F. W., Spencer T. E. (2003). Osteopontin: roles in implantation and placentation. *Biology of Reproduction*.

[41] Liu N., Zhou C., Chen Y., Zhao J. (2013). The involvement of osteopontin and *β*3 integrin in implantation and endometrial receptivity in an early mousepregnancy model. *European Journal of Obstetrics & Gynecology and Reproductive Biology*.

[42] Clifton V. L., Vanderlelie J., Perkins A. V. (2005). Increased anti-oxidant enzyme activity and biological oxidation in placentae of pregnancies complicated by maternal asthma. *Placenta*.

[43] Watanabe H., Hamada H., Yamada N. (2004). Proteome analysis reveals elevated serum levels of clusterin in patients with preeclampsia. *Proteomics*.

[44] Shin J.-K., Han K.-A., Kang M.-Y. (2008). Expression of clusterin in normal and preeclamptic placentas. *Journal of Obstetrics and Gynaecology Research*.

